# Middle Age Has Its Advantages

**DOI:** 10.1371/journal.pgen.1006426

**Published:** 2016-12-01

**Authors:** Maureen M. Barr

**Affiliations:** Department of Genetics and Human Genetics Institute of New Jersey, Rutgers University, Piscataway, New Jersey, United States of America; Washington University School of Medicine, UNITED STATES

If we’re lucky, we get old. Physical and mental decline are negative hallmark associations of aging. Imagine being born blind and, as you approach middle age, waking up with the ability to see. In this issue of *PLOS Genetics*, Cornils, Maurya, and colleagues discover such a phenomenon: age-related suppression of ciliogenesis defects [1].

Cilia are microtubule-based organelles that protrude from most nondividing cells in the human body. Pathologies in cilia formation or function result in human ciliopathies with broad clinical manifestations, including sensory defects, developmental abnormalities, cystic kidneys, infertility, and cancer. Based on the frequency of polycystic kidney disease alone, ciliopathies affect at least 1 in 800 individuals. Hence, identifying a way to recover ciliary function in these patients would have a profound impact.

Our fundamental understanding of ciliogenesis is based on seminal research in *Chlamydomonas* and *Caenorhabditis elegans* [2–6]. Intraflagellar transport (IFT) constructs all cilia and flagella [7]. Anterograde kinesin-2 and retrograde dynein motors transport the multi-protein IFT-A and IFT-B complex machinery and associated cargo along the microtubule-based ciliary axoneme. In *Chlamydomonas* and *C*. *elegans*, mutations in the IFT motors or the IFT machinery result in flagella-less algae or cilia-less worms, respectively. In mice and humans, null mutations in IFT genes are lethal, whereas non-null mutations can produce ciliopathies.

Many of the genes required for the formation, specialization, and sensory properties of *C*. *elegans* cilia have human counterparts that, when mutated, cause ciliopathies [6,8,9]. Unlike mice or humans, worms lacking cilia are viable and fertile under laboratory conditions but exhibit severe sensory defects. Cornils, Maurya, and colleagues performed physicals of middle-aged worms, examining channel cilia of amphid sensory neurons found in the worms’ heads. Shockingly, with age, some previously cilia-less IFT mutants grew functional cilia!

The average life expectancy of a wild-type *C*. *elegans* adult is approximately 12 to 18 days. Age-dependent ciliary recovery (AdCR) of certain IFT mutants occurs between 4 to 7 days of adulthood. AdCR is observed only in hypomorphic, but not null, alleles *of C*. *elegans* IFT-A or IFT-B component genes. These non-null hypomophic alleles include splice site, in-frame deletion, and premature nonsense mutations that have no physiological activity in larval stages and early adulthood. As a consequence, juvenile and young adult mutant animals have stunted and nonfunctional cilia. However, in middle age, IFT-A or IFT-B hypomorphs extend cilia, albeit not to wild-type lengths. Visualization of cilia with in vivo cell-specific reporters reveals AdCR in multiple cilia types, suggesting that, with age, all cilia have the potential to recover from an inherited genetic insult. Ultrastructural analysis using transmission electron microscopy confirms that age-recovered cilia possess both proximal and distal axonemal regions. AdCR is transient—after 7 days of adulthood, cilia are lost.

In wild-type *C*. *elegans* amphid channel cilia, two kinesin-2 motors drive IFT. The slower kinesin-II and faster homodimeric OSM-3/KIF17 act redundantly to construct the proximal microtubule doublet region, whereas OSM-3 acts along to build the distal microtubule singlet region. In middle-aged IFT hypomorphic cilia, kinesin-II moves at a similar rate to that observed in healthy young adult cilia, whereas OSM-3 moves slower at kinesin-II rates, suggesting that kinesin-II but not OSM-3 driving ability is fully restored. Hypomorphic IFT-B and kinesin-II double mutants did not elongate cilia with age, whereas AdCR was observed in hypomorphic IFT-B and *osm-3* double mutants. Based on these data, the authors conclude that AdCR is kinesin-II dependent but not OSM-3 dependent.

AdCR also restores sensory function to IFT-A and IFT-B hypomorphic animals. Severely sensory-deficient animals are now attracted to food sources and avoid harsh osmotic conditions, with increased ciliary length correlating with improved sensory function. Hence, AdCR restores ciliary structure, ciliary motor-based transport, and ciliary physiological functions.

The window of AdCR is further extended in long-lived *daf-2* insulin receptor mutants, consistent with age dependence. In many animal species, reduced insulin signaling increases longevity via activation of the DAF-16/FOXO and HSF-1 transcription factors [10]. Loss of *daf-16* has no impact on AdCR. However, *hsf-1* and *daf-21*/HSP90 heat shock factors are required for AdCR and act cell autonomously. Heat stress does not induce AdCR, indicating that chronic stress is not responsible for revived cilia in aged IFT mutants.

From here, the authors go on to explore potential mechanisms driving AdCR and uncover a role for proteostasis and the ubiquitin-proteasome system (UPS) [11]. In vivo reporters of proteostasis and UPS activity indicate improved proteostasis and increased UPS activity in earlier stages of aging ciliated sensory neurons. Consistent with the UPS being a positive regulator of AdCR, pharmacological inhibition of UPS by bortezomib reduces AdCR.

Bortezomib treatment increases cilia length in wild-type animals, which is the only manipulation Cornils, Maurya, and colleagues found to have an effect in the presence of fully functional IFT machinery. Why UPS inhibition increases cilia length in healthy *C*. *elegans* but blocks AdCR in IFT mutants is unknown. The UPS has been implicated in multiple aspects of cilia biology [12]. For example, increased ubiquitination is observed in disassembling *Chlamydomonas* flagella [13], and the ubiquitin conjugating system is found in both *Chlamydomas* flagellar and mammalian ciliary proteomes [14]. Future studies may provide insight to UPS action in healthy versus mutant cilia and in young versus old cilia.

The paper by Cornils, Maurya, and colleagues is a ground-breaking and pioneering study and opens up an entirely new and unexplored field in cilia cell biology. Moreover, several human ciliopathies are caused by non-null mutations (as null mutations are lethal) or by oligogenic interactions (reducing the function of two or more genes acting in a similar pathway). The authors propose that therapies relieving proteostatic stress may represent a form of personalized medicine for ciliopathy patients with specific mutations in IFT genes.

As with any exciting and unexpected finding, there are more questions than answers. Why is there a critical window for AdCR? What is the mechanism activating AdCR? Is AdCR evolutionarily conserved? Can AdCR be pharmacologically induced in patients? Are there other age-related health improvements and mechanisms yet to be discovered? Cornils, Maurya, and colleagues’ report is encouraging: in some instances, aging can be beneficial.
